# Raw *N*-glycan mass spectrometry imaging data on formalin-fixed mouse kidney

**DOI:** 10.1016/j.dib.2018.08.186

**Published:** 2018-09-12

**Authors:** Ove J.R. Gustafsson, Matthew T. Briggs, Mark R. Condina, Lyron J. Winderbaum, Matthias Pelzing, Shaun R. McColl, Arun V. Everest-Dass, Nicolle H. Packer, Peter Hoffmann

**Affiliations:** a ARC Centre of Excellence in Convergent Bio-Nano Science & Technology, University of South Australia, Mawson Lakes, South Australia 5095, Australia; b Future Industries Institute, University of South Australia, Mawson Lakes, South Australia 5095, Australia; c Adelaide Proteomics Centre, School of Molecular and Biomedical Science, University of Adelaide, Adelaide, South Australia 5005, Australia; d CSL Ltd., Bio21 Institute, Parkville, Victoria 3052, Australia; e Centre for Molecular Pathology, School of Biological Sciences, University of Adelaide, Adelaide, South Australia 5005, Australia; f Australian Institute for Glycomics, Griffith University, Southport, Queensland 4222, Australia; g Biomolecular Discovery and Design Research Centre, Macquarie University, Sydney, New South Wales 2109, Australia

## Abstract

Provided is the annotated raw data for *N*-glycan mass spectrometry imaging (MSI) annotations in thin cross-sections of formalin-fixed and paraffin-embedded murine kidney. Relevant meta-data have been provided in this brief and the raw MSI data can be accessed using ProteomeXchange with the PRoteomics IDEntifications (PRIDE) identifier PXD009808. This brief is the first in a set of submissions from our group which will make raw data publicly accessible for existing and future MSI studies.

**Specifications table**

**Table:** 

Subject area	*Chemistry*
More specific subject area	*Analytical chemistry (mass spectrometry)*
Type of data	*.imzML (imaging)
How data was acquired	*Mass spectrometry (Bruker Daltonics ultrafleXtreme MALDI-ToF/ToF)*
Data format	Raw data (*.imzML)
Experimental factors	*Antigen retrieval, PNGaseF digestion and MALDI matrix deposition*
Experimental features	*Tissue-specific mapping of N-glycan distribution in mouse kidney*
Data source location	*Data was collected at the Adelaide Proteomics Centre (APC) located within the University of Adelaide (South Australia)*
Data accessibility	Raw data (*.imzML) is available on PRIDE (PXD009808*)*

**Value of the data**

• The data supported method development for profiling and MSI of *N*-glycans on FFPE tissue sections.
• The data is linked to orthogonal methods for derivatization and LC–MS/MS identification of *N*-glycan composition.
• Other MSI researchers are now able to view, process and further analyze this *N*-glycan data.

## 1 Data

Prior to this data-brief the *N*-glycan MSI data [1] was made partially available as an upload to SCiLS CLoud. This provided a tool for online visualization of the spatial distribution for a selection of ions detected from the FFPE murine kidney sections used in these experiments – and limited figure or ion map downloads. The selectable ions included both a set of 18 *N*-glycan candidates and a filtered set of 203 *m*/*z* intervals (Signal to Noise Ratio (SNR) > 3 and *m/z* > 933), all of which can be visualized using multiple signal normalization approaches, including Root Mean Square (RMS), Total Ion Count (TIC) or median. The SCiLS CLoud availability of the MSI data was a step in the right direction. To complete the transparent dissemination of this data it was uploaded to ProteomeXchange Consortium [2] via the PRIDE partner repository [3] in the community standard *.imzML format as a partial submission: the data is available at the following link (https://www.ebi.ac.uk/pride/archive/projects/PXD009808) with the dataset identifier PXD009808.

## 2 Experimental design, materials and methods

**Fig. 1:**
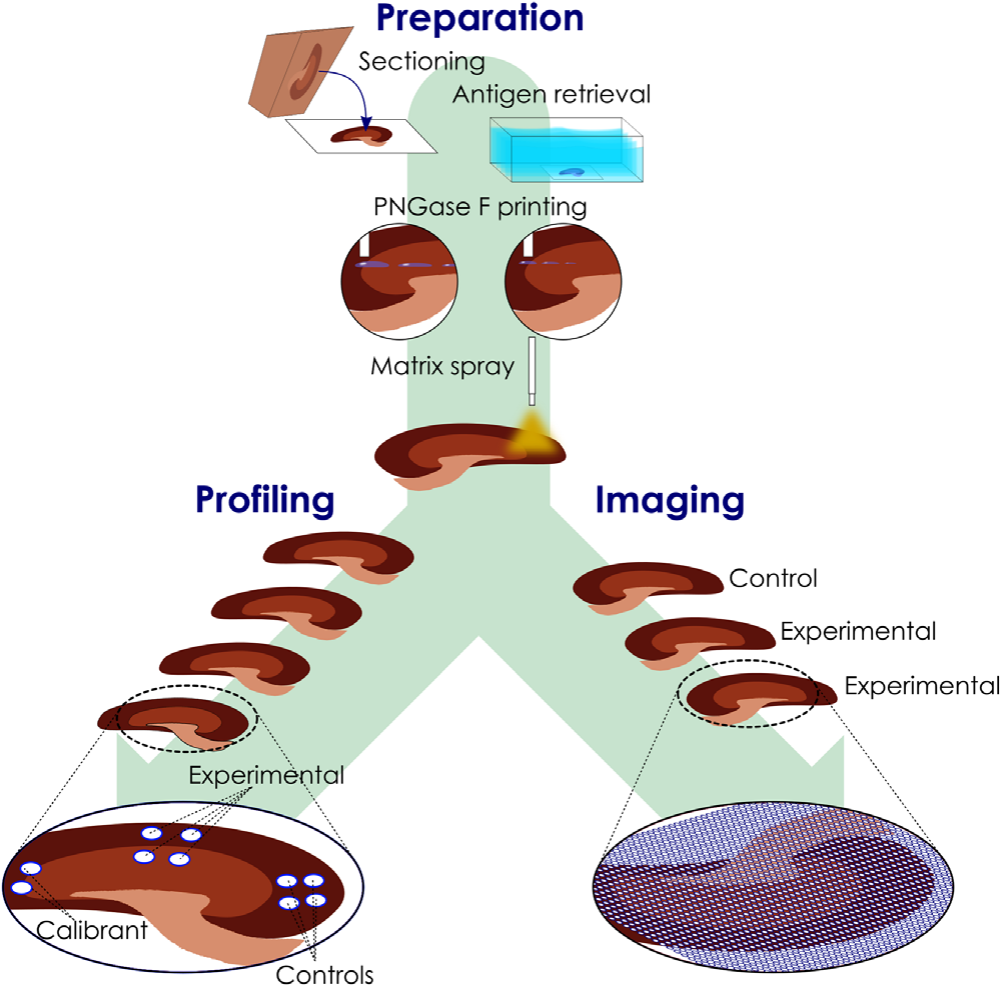
*N-*glycan profiling MS and MSI workflow.

The materials used, as well as the supplier, are provided in the tables below. For clarity these are split between chemicals (Table 1), mass standards (Table 2) and equipment/consumables (Table 3). The experimental design and methods are described below. The data acquisition was split by the experimental design into two aspects: profiling MS and MSI (see Fig. 1).

1. *Profiling* of specific kidney tissue regions using high volume (750 nL) printed PNGase F deposited at a centre to centre spacing of 1300 µm. This also incorporated control 25 mM Ammonium Bicarbonate (NH_4_HCO_3_) buffer-only spots (see Fig. 1).

**Table:** 

*Number of sections*	4
PNGase F replicate spots (total)	2/tissue region (*N* = 4)
Control replicate spots	4 replicate spots in kidney cortex
2. *MSI* using 30 nL printed PNGase F deposited in an array of spots with a centre to centre spacing of 250 µm.

**Table:** 

*Number of sections*	3
PNGase F regions (total)	1 per kidney, complete section (*N* = 2)
Control regions	1 (half kidney section)

**Table 1:** List of chemicals and suppliers.

*Item*	*Details (supplier)*
Glycerol-free PNGase F	P0705L, 75000 NEB units (New England BioLabs, Ipswich, MA, USA)
Formalin	Sigma-Aldrich
Trifluoroacetic acid (TFA)	Merck (Darmstadt, Germany)
Ethanol (EtOH)	Merck (Darmstadt, Germany)
Sodium chloride (NaCl)	Merck (Darmstadt, Germany)
2,5-dihydroxybenzoic acid (DHB)	Sigma-Aldrich/Bruker Daltonics (Bremen, Germany)
Xylene	Chem-Supply (Gillman, South Australia)

**Table 2:** Mass standards used to externally calibrate mass spectrometry data.

*Item*	*Supplier*	*m/z*
Man5GlcNAc2	Prozyme (CA, USA)	[M+Na]+: 1257.4225
Man3GlcNAc5	Prozyme (CA, USA)	[M+Na]+: 1542.5551
Man3Gal4GlcNAc6	Prozyme (CA, USA)	[M+Na]+: 2393.8457

**Table 3:** Consumables and equipment, including suppliers.

*Item*	*Supplier*
Indium tin oxide (ITO) slides	Bruker Daltonics (Bremen, Germany)
Poly Ethylene Naphthalate (PEN) slides	MicroDissect (Herborn, Germany)
0.025 µm VWSP nitrocellulose membranes	Millipore (Cork, Ireland)
TP 1020 processors	Leica Biosystems (North Ryde, Australia)
EG 114OH embedder	Leica Biosystems
Microm HM 325 microtome	Zeiss (Gottingen, Germany)
TM-sprayer	HTX Instruments (NC, USA)
UltrafleXtreme MALDI-ToF/ToF	Bruker Daltonics
Chemical Inkjet Printer (ChIP)-1000	Shimadzu (Japan)

For both the profiling and MSI modes, the PNGase F was deposited using a Chemical InkJet Printer 1000 (ChIP-1000) instrument. Following incubation to allow the enzyme to cleave *N*-glycans from the fixed tissue the sections were overlaid with 2,5-dihydroxybenzoic acid (DHB) in 1 mM sodium chloride (NaCl) and 0.1% trifluoroacetic acid (TFA) using a capillary nebulized spray delivered via a TM-sprayer instrument.
